# The associations of stroke, transient ischemic attack, and/or stroke-related recurrent vascular events with Lipoprotein-associated phospholipase A_2_: A systematic review and meta-analysis

**DOI:** 10.1097/MD.0000000000009413

**Published:** 2017-12-22

**Authors:** Ye Tian, Huan Jia, Sichen Li, Yanmin Wu, Li Guo, Guojun Tan, Bin Li

**Affiliations:** a Department of Neurology, The Second Hospital of Hebei Medical University; b Key Laboratory of Hebei Neurology, Shijiazhuang, Hebei, China.

**Keywords:** ischemic stroke, lipoprotein-associated phospholipase A_2_, meta-analysis, recurrent vascular event, stroke, systematic review, TIA

## Abstract

**Background:**

Studies on stroke and lipoprotein-associated phospholipase A_2_ (Lp-PLA_2_) have produced conflicting results.

**Objective:**

The aim of the study was to assess the associations of Lp-PLA_2_ levels (mass and activity) with recurrent vascular events in patients with transient ischemic attack (TIA) and/or first ischemic stroke and with stroke in the general population.

**Methods:**

The MEDLINE, Embase, the Cochrane Library, Web of Science, Science Direct, China National Knowledge Infrastructure, China Biology Medical Disc (CBMdisc), and WanFang were searched for prospective observational studies reported until January 2017. Eligible studies reported Lp-PLA2 levels and adjusted risk estimates of recurrent vascular events and/or stroke. Risk ratio (RR) with corresponding 95% confidence intervals (CIs) were used to express the pooled data in a random-effects model.

**Results:**

A total of 11 studies that comprised 20,284 participants (4,045 were TIA and/or first ischemic stroke patients and 16,239 were residents in general population) were identified, which reported either Lp-PLA_2_ mass levels (4 studies) or Lp-PLA_2_ activity levels (10 studies). The pooled RR of recurrent vascular events (467 cases) in TIA and/or first ischemic group was 2.24 (95% CI, 1.33–3.78), whereas the pooled RR of stroke (1604 cases) in the general population was 1.47 (95% CI, 1.10–1.97). The pooled RRs of Lp-PLA_2_ mass and activity levels with the risk of stroke in the general population were 1.69 (95% CI, 1.03–2.79) and 1.28 (95% CI, 0.88–1.85), respectively.

**Conclusions:**

In patients with TIA and first ischemic stroke, elevated Lp-PLA2 activity levels were associated with recurrent vascular events. And in the general population elevated Lp-PLA2 levels were associated with the risk of stroke, although the association between Lp-PLA_2_ activity levels and the risk of stroke was less profound compared with the corresponding association of stroke risk with the Lp-PLA_2_ mass levels.

## 1 Introduction

Stroke is considered as the main cause of death and neurologic disability worldwide. A considerably high number of cases worldwide are reported to suffer from stroke and transient ischemic attack (TIA) annually, of which more than one-fifth will experience a recurrent stroke within a short period of time.^[1]^ Management of vascular risk factors is of great importance for primary and secondary prevention of stroke.^[2,3]^ As conventional risk factors such as hyperglycemia, hyperlipidemia, and blood pressure are inadequate in predicting stroke,^[4,5]^ novel predictive biomarkers were investigated to predict high-risk subjects.^[6,7]^

Approximately 87% of all stroke incidents are ischemic stroke incidents,^[8]^ most of which are highly associated with the etiology of atherosclerosis. Inflammation is postulated to play a significant role in the process of atherosclerosis.^[9]^ Lipoprotein-associated phospholipase A2 (Lp-PLA2) is an enzyme derived from inflammatory cells that is mainly bound to low-density lipoproteins (LDL) in the circulation.^[10]^ Lp-PLA2 has been reported to be associated with atherosclerotic plaque inflammation and instability.^[11]^ Lp-PLA2 plays a role in hydrolysis of oxidized LDLs by the production of proinflammatory mediators, namely, lysophosphatidylcholine and oxidized nonesterified fatty acids that are involved in the migration of vascular smooth muscle cells, endothelial dysfunction, expression of adhesion molecules and cytokines, as well as the formation of necrotic core in plaques.^[12–14]^

A majority of studies have confirmed the relationship between Lp-PLA2 mass and/or activity levels and the risk of subsequent cardiovascular diseases (CVD), but investigations of Lp-PLA2 mass and/or activity levels and the risk of stroke have produced conflicting results. The available data in a previous review indicated that the determination of Lp-PLA2 levels was associated with the risk of stroke risk which approximately doubled the increase noted in the occurrence of stroke.^[15]^ A meta-analysis of 32 prospective studies suggested that circulating Lp-PLA2 mass and activity levels demonstrated an association with the risk of coronary heart disease, but the association of Lp-PLA2 was less evident with regard to ischemic stroke.^[16]^ Certain studies further included stroke as a combined endpoint^[17,18]^ although a single meta-analysis of the risk of TIA and stroke was not conducted.

The objective of the present meta-analysis was to assess the available evidence of associations of Lp-PLA2 levels (mass and activity) with TIA and/or stroke-related recurrent vascular events and with the incidence of stroke in the general population, respectively.

## 2 Methods

This study was conducted in accordance with the Preferred Reporting Items for Systematic Reviews and Meta-analyses (PRISMA) statement.^[19]^ All analyses were based on previous published studies, thus no ethical approval and patient consent was required.

### 2.1 Search strategy

The following databases were used as online sources: MEDLINE, Embase, the Cochrane Library, Web of Science, Science Direct, China National Knowledge Infrastructure, China Biology Medical Disc (CBMdisc), and WanFang, and the time period included the initial inspection till January 2017. The languages of the studies investigated were restricted to English and Chinese. The search terms and their related combinations were the following: “lipoprotein-associated phospholipase A2” OR “Lp-PLA_2_” OR “Lp-PLA_2_ and platelet-activating factor acetylhydrolase” OR “PAF acetylhydrolase” AND “Stroke” OR “ischemic stroke” OR “hemorrhagic stroke” OR “cerebrovascular disease” OR “brain infarction” OR “intracranial vascular disorder” OR “apoplexy” OR “cerebrovascular insufficiency” OR “cerebrovascular accident” OR “transient ischemic attack” OR “TIA” OR “recurrent vascular event” OR “recurrent stroke.” The reference lists of all selected studies, conference abstracts, and reviews were further manually screened to supplement for eligible studies.

### 2.2 Study selection

Studies were considered eligible if they accorded with the following criteria: the present study was a prospective observational study. Participants with TIA/first ischemic stroke were classified into recurrent group and participants from general population were allocated to the stroke group. Blood levels of Lp-PLA2 mass and/or activity were measured. Adjusted hazard ratio (HR) and risk ratio (RR) with 95% confidence intervals (CIs) of recurrent vascular events and/or stroke were reported with a cutoff value, defined as the highest quantile vesus the bottom quantile and/or per 1 SD change in Lp-PLA2 levels. Reviews, editorials, case reports, correspondences, experimental studies, and studies with <50 participants were excluded. Studies that were published with similar population sizes were selected based on the most complete presentation of data and the date of publication.

### 2.3 Data extraction

Data from eligible studies were extracted by 2 independent reviewers (Ye Tian and Huan Jia) using the same standard extraction form. Certain discrepancies were resolved by discussion and/or consulting to a senior author (Bin Li). The variables extracted from eligible studies were the following: first author's name, publication date, original country, study design, sampling frame, number of participants, mean age and/or age range, endpoint event, measurement of Lp-PLA_2_, fully adjusted risk estimates and corresponding CIs, follow-up duration, and adjusted covariates. Some of the authors were contacted for information that was unclear and data that were unpublished. Stroke was defined as a focal neurological defect confirmed by physical examination and CT scan and/or magnetic resonance imaging (MRI), which lasted >24 hours with a rapid onset.^[20]^ Recurrent vascular events included TIA, ischemic and/or hemorrhage stroke, myocardial infarction, and vascular death.

### 2.4 Quality assessment

The methodological qualities of included studies were assessed according to the Newcastle-Ottawa Scale (NOS), which is commonly used to evaluate the quality of nonrandomized studies.^[21]^ A total of 3 domains were judged in NOS, including the selection of the subjects, the comparability of groups, and the assessment of exposure and outcomes. Each study was evaluated using a scoring system of 0 to 9 stars, based on the aforementioned 3 domains. The studies that achieved 6 or more stars were regarded as high quality studies.

### 2.5 Statistical analyses

The present meta-analysis was conduced using STATA (version 12.0 College Station, TX; StataCorp LP). A *P* < .05 was considered statistically significant. The variables adjusted HR and RR with 95% CIs were further used to calculate the pooled risk estimates. In addition, pooled risk ratios were used as risk estimates due to the relatively low morbidity of the diseases investigated and the similarity in the interpretation of these parameters. The Cochran *Q* tests and *I*^2^ statistics were used to examine the heterogeneity between studies. A random-effects model was used if *P* was <.1 in *Q* tests and/or at *I*^2^ score of 50% or higher, which indicated larger heterogeneity across studies. A fixed-effects model was used for the remaining studies that deviated from these cutoff values.^[22]^ Subgroup analyses were carried out to compare recurrent vascular events in TIA and/or primary ischemic stroke and the different types of Lp-PLA_2_ assays with the risk of stroke in the general population. Sensitivity analysis was carried out to identity the study responsible for the heterogeneity and/or to test the validity of the conclusions by omitting one study at sequentially. Begg rank correlation and Egger linear regression tests were used to screen for the potential publication bias. When the *P* value was >.05 in the combined Begg and Egger tests, the publication bias was regarded as absent.

## 3 Results

### 3.1 Literature search

**Figure 1:**
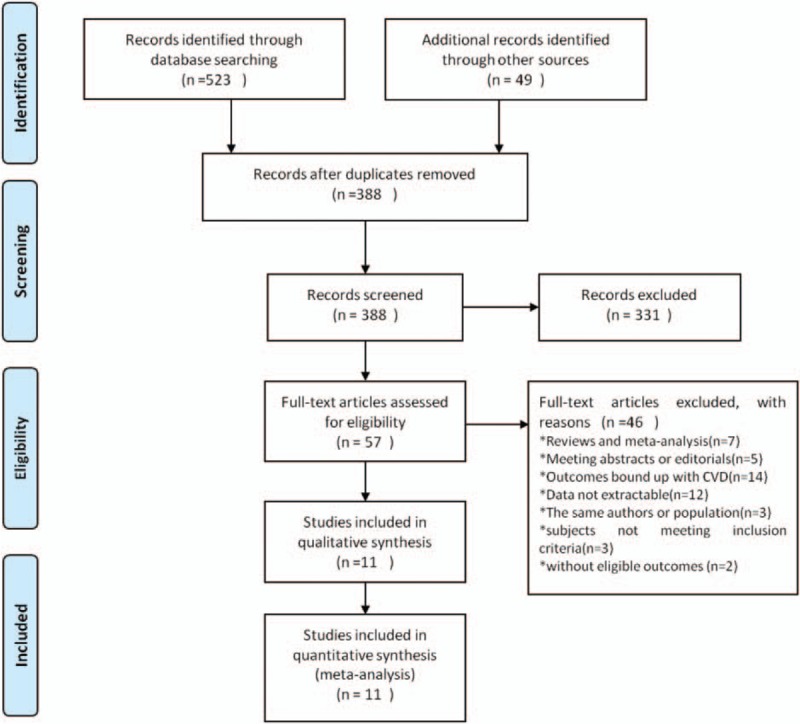
Flow diagram of study selection.

A total number of 572 studies were identified via electronic database search. After omission of duplicated studies, the titles and abstracts of 338 studies were screened. A further assessment of 57 full-text articles and 16 studies was in alignment with the inclusion criteria. A total of 5 articles were further removed and manual examination of reference lists did not produce additional articles for evaluation. Eventually 11 studies^[23–33]^ were included in the final analysis (Fig. 1).

### 3.2 Characteristics of eligible studies and quality assessment

The characteristics of the eligible studies are summarized in Table 1. A total of 20,284 participants were included in 11 studies, of which 4,045 were TIA and/or first ischemic stroke patients reported in 5 studies^[33,26–29]^ and 16,239 were residents in the general population reported in 6 studies.^[24,25,30–33]^ Within all eligible studies, 3 were prospective case–cohort studies,^[25,30,31]^ whereas the remaining 8 were prospective cohort studies.^[23,24,26–29,32,33]^ The sample size ranged from 75 to 5393 and the follow-up duration period varied from 30 days to 11 years. A total of 4 studies reported Lp-PLA_2_ mass levels,^[24,25,30,33]^ whereas 10 studies reported Lp-PLA_2_ activity levels.^[23–29,31–33]^ A total of 3 studies included results of both Lp-PLA_2_ assays.^[24,25,33]^ In addition, 2 studies further reported the results of the etiological classification of stroke.^[24,26]^

**Table 1:**
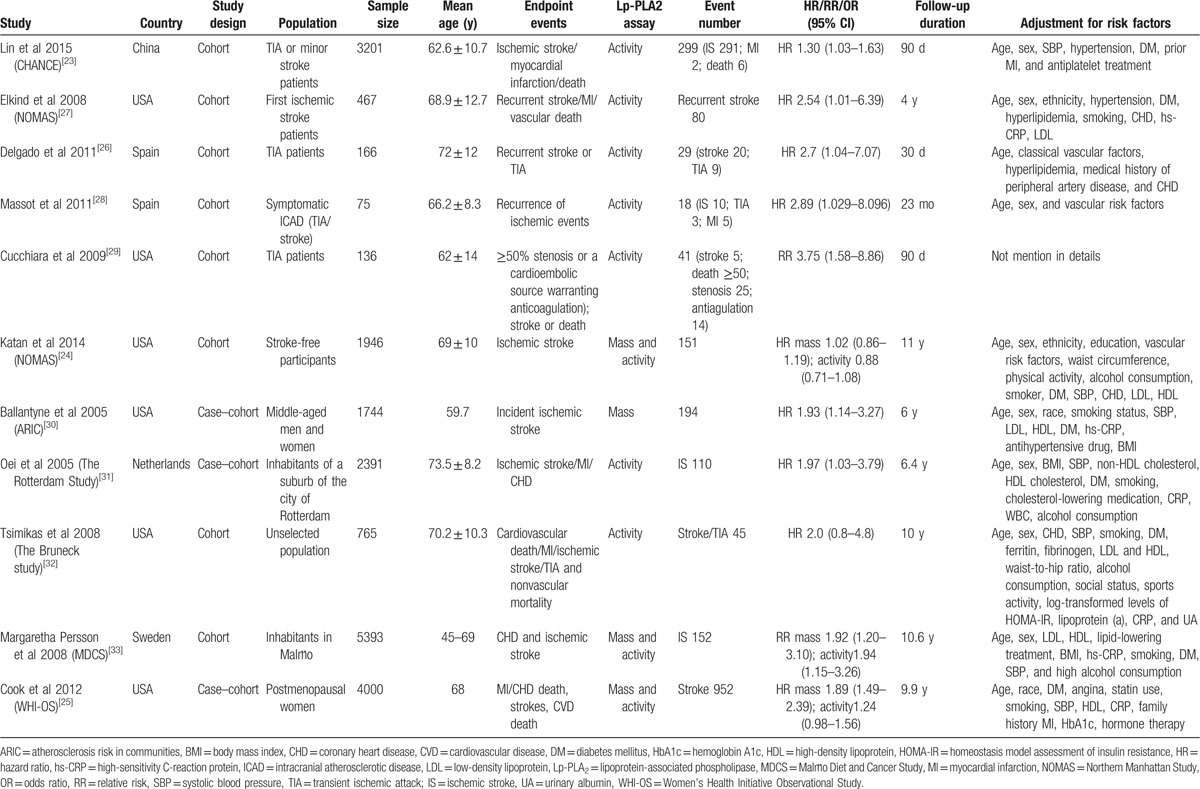
Characteristics of included studies in the meta-analysis.

The methodological qualities of the included studies are shown in Table 2 with an acceptable range of results. The qualities of all eligible studies included in the present analysis were between moderate to high and the score ranged from 5 to 9 stars according to the NOS.

**Table 2:**
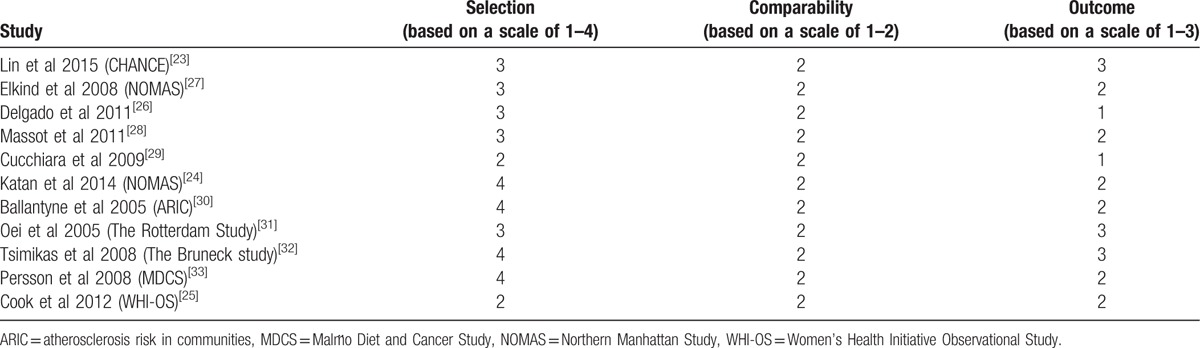
Quality assessment of eligible studies.

### 3.3 Lp-PLA_2_ activity is associated with the risk of TIA and/or stroke-related recurrent vascular events

**Figure 2:**
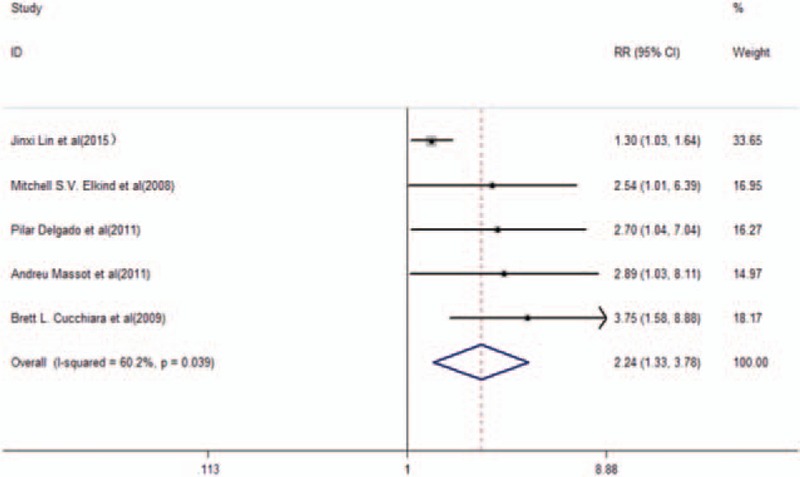
RR and 95% CI of Lp-PLA_2_ activity levels and recurrent vascular events in TIA/primary ischemic stroke patients in a random-effects model.

**Figure 3:**
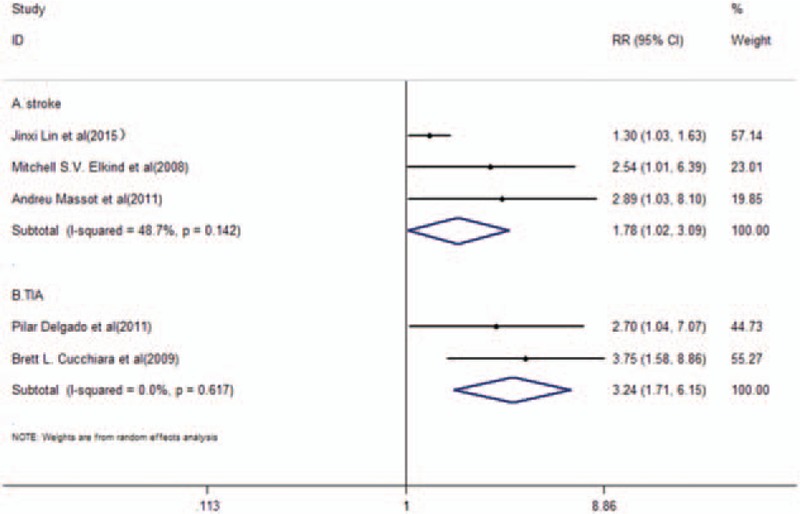
RR and 95% CI of Lp-PLA_2_ activity levels and recurrent vascular events in stroke patients (A) or TIA patients (B) in a random-effects model.

Patients with TIA and/or primary ischemic stroke at baseline were reported in 5 studies.^[23,26–29]^ A total number of 467 recurrent vascular events that were related to TIA and/or stroke occurred in 4045 participants. The pooled RR of further adjustment was 2.24 (95% CI, 1.33–3.78, *P* = .002) with high heterogeneity (*I*^2^ = 60.2%, *P* = .039). Furthermore, a random-effects model was used (Fig. 2). Subgroup analysis was conducted to compare the recurrent vascular events in TIA patients with the incidence of stroke. A total of 3 studies^[23,27,28]^ that included patients with stroke exhibited further adjustment of pooled RR of 1.78 (95% CI, 1.02–3.09, *P* = .042) with moderate score (*I*^2^ = 48.7%; *P* = .142) in a random-effects model (Fig. 3A). Patients with TIA were solely reported in 2 studies^[26,29]^ (Fig. 3B) with a pooled RR of further adjustment of 3.24 (95% CI, 1.71–6.15, *P* < .001) and no evidence of heterogeneity (*I*^2^ = 0, *P* = .617).

### 3.4 Lp-PLA_2_ levels were associated with the risk of stroke

**Figure 4:**
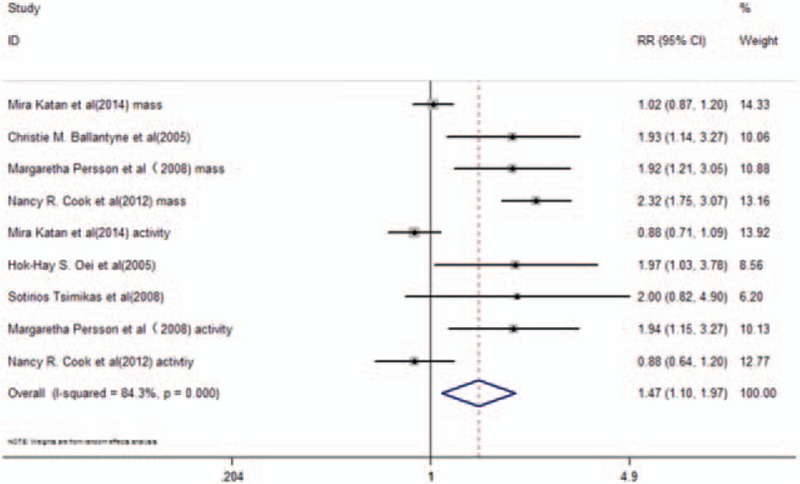
RR and 95% CI of Lp-PLA_2_ levels and the risk of stroke in the general population in a random-effects model.

**Figure 5:**
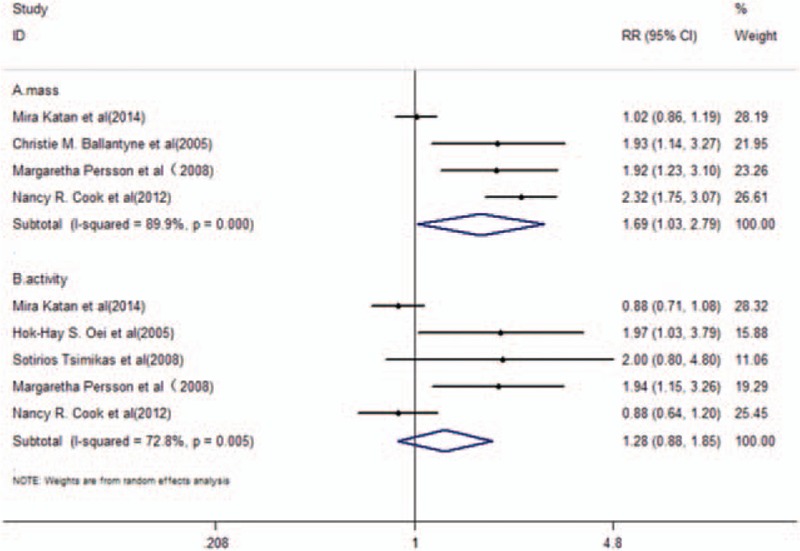
RR and 95% CI of Lp-PLA_2_ mass (A) or activity (B) levels and the risk of stroke in the general population in a random-effects model.

Stroke was reported as an endpoint in the general population in 6 studies^[24,25,30–33]^ that further included the determination of Lp-PLA_2_ mass and activity levels. A total of 16,239 participants were included in the meta-analysis, of which 1,604 were stroke cases. The pooled RR of further adjustment was 1.47 (95% CI, 1.10–1.97, *P* = .01) with high heterogeneity (*I*^2^ = 84.3%, *P* < .001) in a random-effects model (Fig. 4). Subgroup analysis was carried out based on the different assays (mass and/or activity levels) of Lp-PLA_2_. A total of 4 studies^[24,25,30,33]^ that included reported Lp-PLA_2_ mass levels revealed pooled RR of further adjustment to 1.69 (95% CI, 1.03–2.79, *P* = .039) with high heterogeneity (*I*^2^ = 89.9%; *P* < .001) in a random-effects model (Fig. 5A). Lp-PLA_2_ activity levels were reported in 5 included studies of this group^[24,25,31–33]^ (Fig. 5B). The pooled RR of further adjustment was 1.28 (95% CI, 0.88–1.85, *P* = .198) with high heterogeneity (*I*^2^ = 72.8%; *P* = .005) in a random-effects model (Fig. 5B).

### 3.5 Sensitivity analyses and publication bias assessment

Significant heterogeneity was observed with a random-effects model. Sensitivity analysis was conducted by sequential omission of one study. The results of the sensitivity analyses demonstrated influences in the quantitative pooled estimates of RR and its 95% CI, and heterogeneity between the different studies examined. The heterogeneity decreased considerably (*I*^2^ = 0%, *P* = .934) for patients with TIA and/or stroke and the pooled adjusted RR was increased from 2.24 (95% CI, 1.33–3.78, *P* = .002) to 2.97 (95% CI, 1.86–4.75, *P* < .001) provided that one study^[23]^ was excluded. The heterogeneity of patients from the general population was considerably altered (*I*^2^ = 0%, *P* = .714) between the studies that used the Lp-PLA_2_ mass assay determination after omission of one study.^[24]^ Furthermore, the pooled adjusted RR changed from 1.69 (95% CI, 1.03–2.79, *P* = .039) to 2.15 (95% CI, 1.73–2.68, *P* < .001), whereas a minor change was noted between the studies that used the Lp-PLA_2_ activity assay determination.

**Figure 6:**
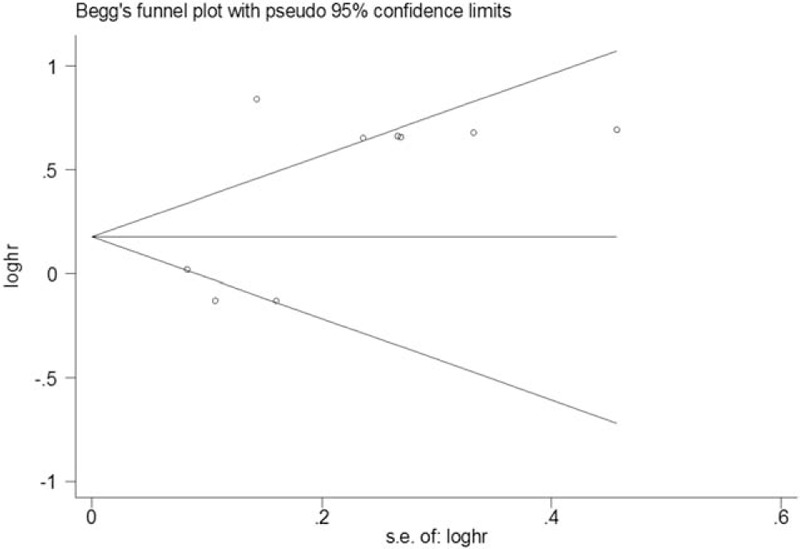
Begg funnel plot of studies in the general population.

**Figure 7:**
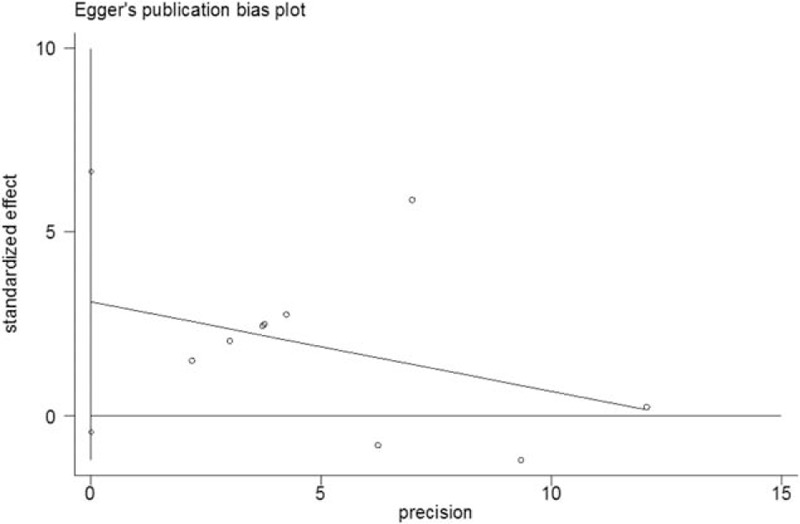
Egger publication bias of studies in the general population.

No significant publication bias was noted within included studies of participants from the general population, as indicated by both Begg (*P* = .917) and Egger (*P* = .077) tests (Figs. 6 and 7).

## 4 Discussion

The results of the present meta-analysis indicated that elevated blood levels of Lp-PLA2 activity were associated with increased risk of TIA/primary ischemic stroke-related recurrent vascular events. Higher blood mass levels of Lp-PLA2 were associated with the risk of stroke in the general population, whereas the association of blood Lp-PLA2 activity levels with stroke was less evident compared with that noted regarding Lp-PLA2 mass and the risk of stroke.

The findings of the present study indicated that patients with TIA and/or primary ischemic stroke who exhibited elevated blood levels of Lp-PLA_2_ activity were at higher risk of developing recurrent vascular events. A specific study^[23]^ included in the analysis may be responsible for the heterogeneity in this group. This study was a prospective study focused on Asian subjects that measured Lp-PLA_2_ activity with an automated enzyme assay system on a Hitachi 7600 analyzer. The differences in the study design, race, and Lp-PLA_2_ activity measurement may directly influence the results. Moreover, Stafforini et al demonstrated that loss-of-function mutations in the PLA2G7 gene are common in East-Asian populations, which could effectively abolish Lp-PLA_2_ activity and/or largely reduce the activity levels in heterozygotes.^[34]^ Subgroup analysis indicated that Lp-PLA_2_ activity levels in patients with TIA were associated with a higher risk of recurrent vascular events compared with patients with stroke. A study revealed higher expression of Lp-PLA_2_ and its products in carotid plaques from symptomatic patients with TIA and ischemic stroke compared with asymptomatic patients. It is interesting to note that in the symptomatic group TIA patients seemed to be responsible for the observed differences and showed the highest expression of Lp-PLA_2_.^[35]^ The present study was notably based on a small number of studies. Consequently, the possibility of coincidence and the contribution of other confounding factors cannot be excluded and more studies are required to confirm the current findings.

The results of the current meta-analysis indicated that elevated blood levels of Lp-PLA_2_ mass were associated with the risk of stroke in the general population. A limited number of systematic reviews and meta-analyses supported the relationship between circulatory Lp-PLA_2_ levels and the risk of CVD. The majority of these studies included stroke in the list of combined endpoints.^[16–18]^ A total of 2 main assays were used in the included studies to measure Lp-PLA_2_ levels, one corresponding to the determination of the mass levels and the other to the measurement of the activity levels. The correlations of these 2 assays varied in different studies, ranging from *r* = 0.36 in the PROVEIT trial^[36]^ to *r* = 0.89 in a smaller study that included solely men,^[37]^ which could possibly influence the results. Subgroup analysis was carried out to compare the different assays of Lp-PLA_2_ levels with the risk of stroke in the general population.

The relationship between elevated blood levels of Lp-PLA_2_ mass and the risk of stroke was observed in the present meta-analysis with evidence of heterogeneity. The results of the sensitivity analyses demonstrated that in the case of omitting one of the included studies, the heterogeneity would disappear. One possible explanation for this finding was the ethnic difference that influenced the results. The majority of the participants in the present study^[24]^ were of Hispanic origin, whereas the populations examined in the majority of the other studies in this group were of white origin. Although Katan et al demonstrated that Lp-PLA_2_ mass levels were not associated with overall ischemic stroke, it was suggested that the corresponding levels were associated with the risk of atherosclerotic stroke among non-Hispanic white participants,^[24]^ which partly supported the results of our findings.

Contrary to Lp-PLA_2_ mass levels, the elevated levels of Lp-PLA_2_ activity seemed not to significantly affect the incidence of stroke after further adjustment for risk factors. The present study supported the results of a former collaborative analysis,^[16]^ which documented the association of Lp-PLA_2_ protein and activity levels with the levels of proatherogenic lipids after adjustment for conventional risk factors (RRs) that were 1.14 (95% CI, 1.02–1.27) and 1.08 (95% CI, 0.97–1.20) for ischemic stroke, respectively. Certain studies indicated that Lp-PLA_2_ activity levels exhibited a higher association with the expression of multiple lipid markers, namely, high-density lipoproteins (HDL) and LDL cholesterol compared with Lp-PLA_2_ protein levels.^[25,37–39]^ This finding could indicate differences after various adjustments and differences in the measurement precision. Furthermore, the unreliable correction for regression dilution during long-term follow-up in prospective studies may underestimate risk associations.^[40]^ In addition, in case of adjusted analyses, the specific information regarding certain potential confounding factors, such as medication for vascular diseases, was not uniformly obtained from included studies.^[16]^ This discrepancy further affected the results obtained in the present analysis.

The majority of the included studies focused mainly on the relationship between Lp-PLA_2_ mass and/or activity levels and ischemic stroke. Clinical data suggested that the elevated levels of Lp-PLA_2_ mass and the higher levels of the corresponding activity were associated with the progression of atherosclerotic disease.^[41]^ Certain human- and animal-based studies further highlighted an increase in the expression of Lp-PLA_2_ in atherosclerotic lesions and plasma, which were related to accelerated atherogenesis.^[35,42,43]^ Although the most common cause of ischemic stroke is atherosclerosis, the causes of ischemic stroke are more heterogeneous compared with those noted in atherosclerotic heart disease. Subsequently, the effects of particular risk factors may be underestimated considering all types of ischemic stroke as one. One included study indicated that Lp-PLA_2_ mass levels were associated with the risk of atherosclerotic stroke among non-Hispanic white participants,^[24]^ whereas other studies have shown that Lp-PLA_2_ activity levels were increased significantly when considering a large-artery atherosclerotic (LAA) etiology as the most likely mechanism for the TIA incidence.^[26]^ It is tempting to speculate that due to the limited outcomes recorded, there is insufficient specific evidence regarding the relationship between Lp-PLA_2_ levels and the etiological classifications of ischemic stroke. Thus, further studies are required to provide more information on this topic.

The potential advantages and limitations of the current systematic review should be taken into consideration. Instead of mainly focusing on Lp-PLA_2_ levels and CVD as shown by previous meta-analyses,^[16,17]^ we emphasized on the relationship between Lp-PLA_2_ mass and/or activity levels and the incidence of recurrent vascular events and/or primary stroke events, respectively. However, the limited number of included studies was not sufficient to eliminate a part of the heterogeneity observed. In addition, the restricted access to the full spectrum of data required for the study analysis was a significant limitation of the present study. Second, most of the Lp-PLA_2_ levels were measured at a single time point rather than conducting serial measurements, which could not correct for the regression dilution.^[40]^ Third, studies included were not based on random controlled trails (RCTs), the power of the results might be decreased to some extent, and certain potential confounding factors could not uniformly be adjusted in individual studies, which might lead to an overestimation and/or underestimation of the risk. In addition, the study was based on published data and the publication bias might have a significant effect on the results interpretation. Consequently, unpublished data should also be included to reduce the effect of selective reporting. The aforementioned limitations should be addressed by larger well-designed studies focusing on the relationship between serial measurements of Lp-PLA_2_ levels and the risk of stroke subtypes. In addition, well-designed studies are required to be conducted that will explore the association between Lp-PLA_2_ levels and stroke with regard to the ethnic origin of the sample population, notably between Asian and European subjects.

The present meta-analysis suggested that blood Lp-PLA_2_ activity levels could potentially be used as a predictor of recurrent vascular events in patients with TIA or first ischemic stroke. Furthermore, Lp-PLA_2_ mass levels could be used for stroke risk stratification in the general population. Lp-PLA_2_ can be regarded as a therapeutic target in the prevention of stroke and random trails of potent pharmacological inhibitors should be conducted.

Elevated blood levels of Lp-PLA_2_ activity were associated with increased risk of recurrent vascular events in patients with TIA or primary ischemic stroke, whereas elevated Lp-PLA_2_ mass levels were related to the risk of stroke in the general population. The relationship between Lp-PLA_2_ activity levels and stroke was less profound compared with the relationship noted between the Lp-PLA_2_ mass levels and the risk of stroke. Further well-designed studies are required to update and confirm the findings presented in the current meta-analysis.
